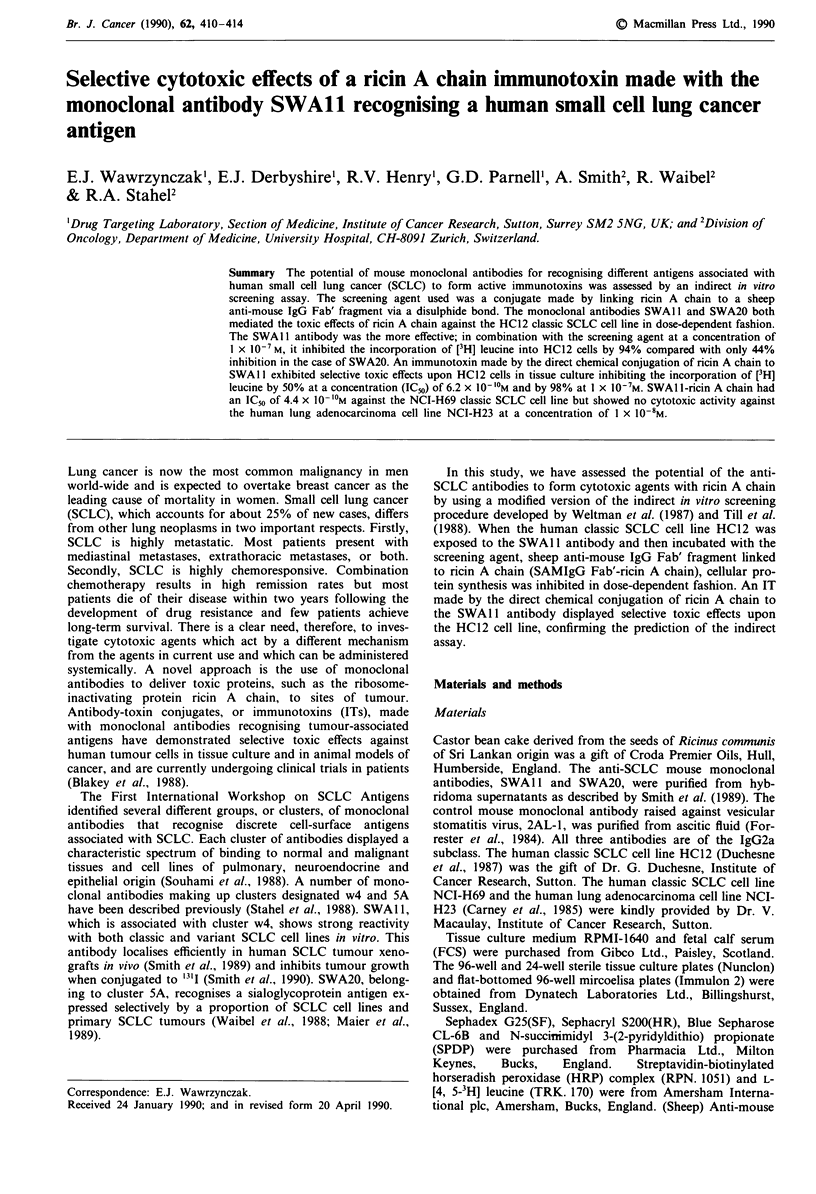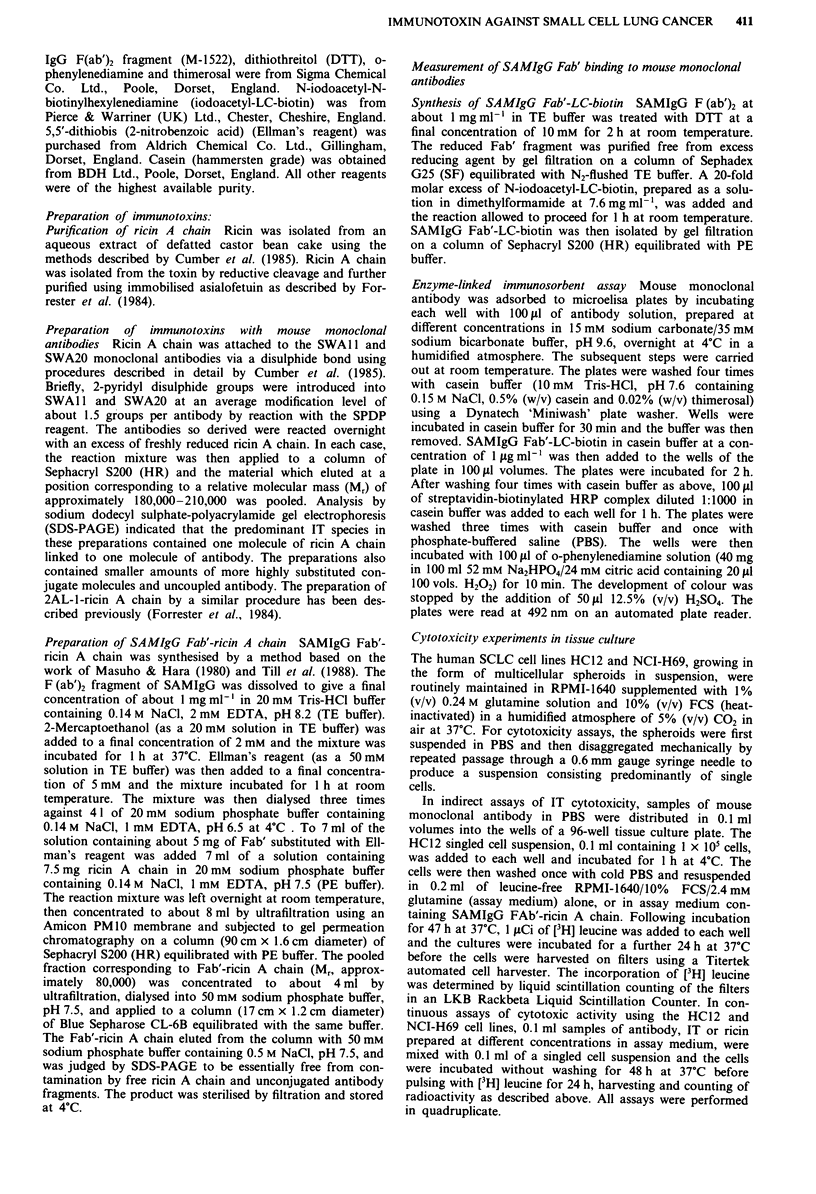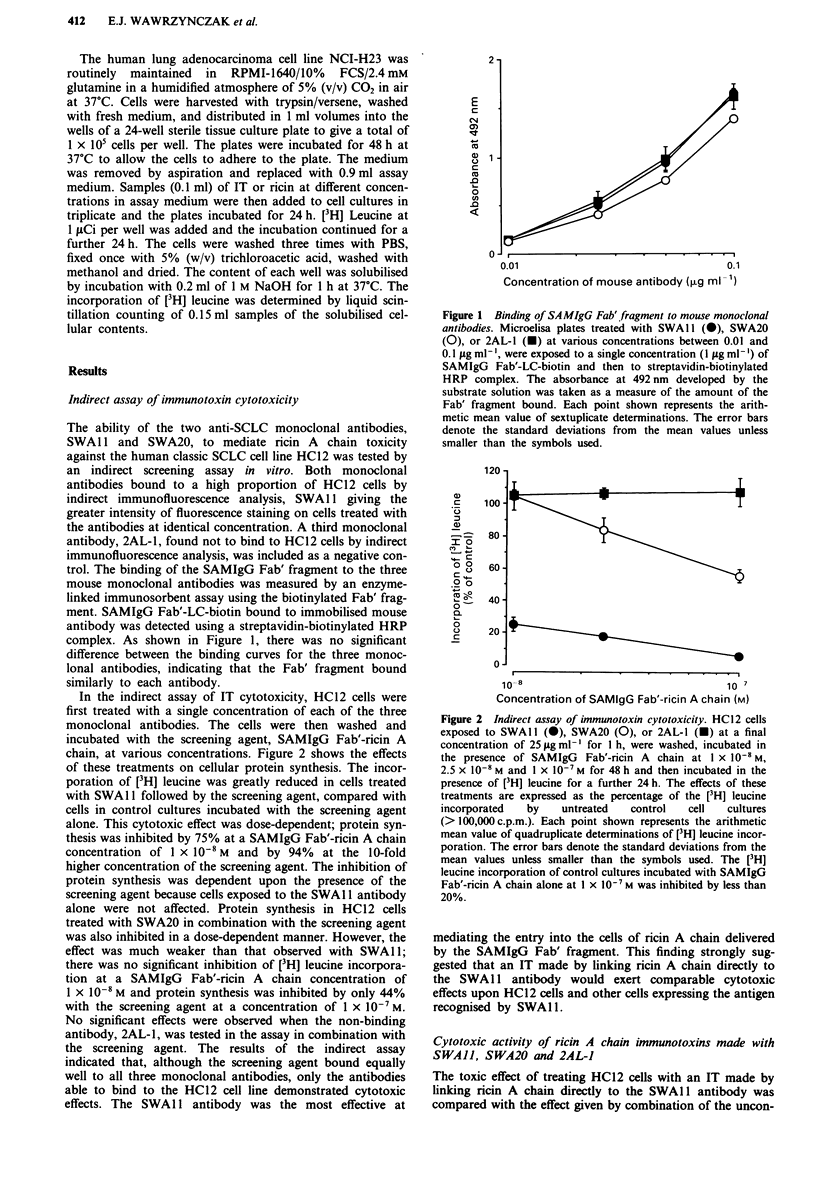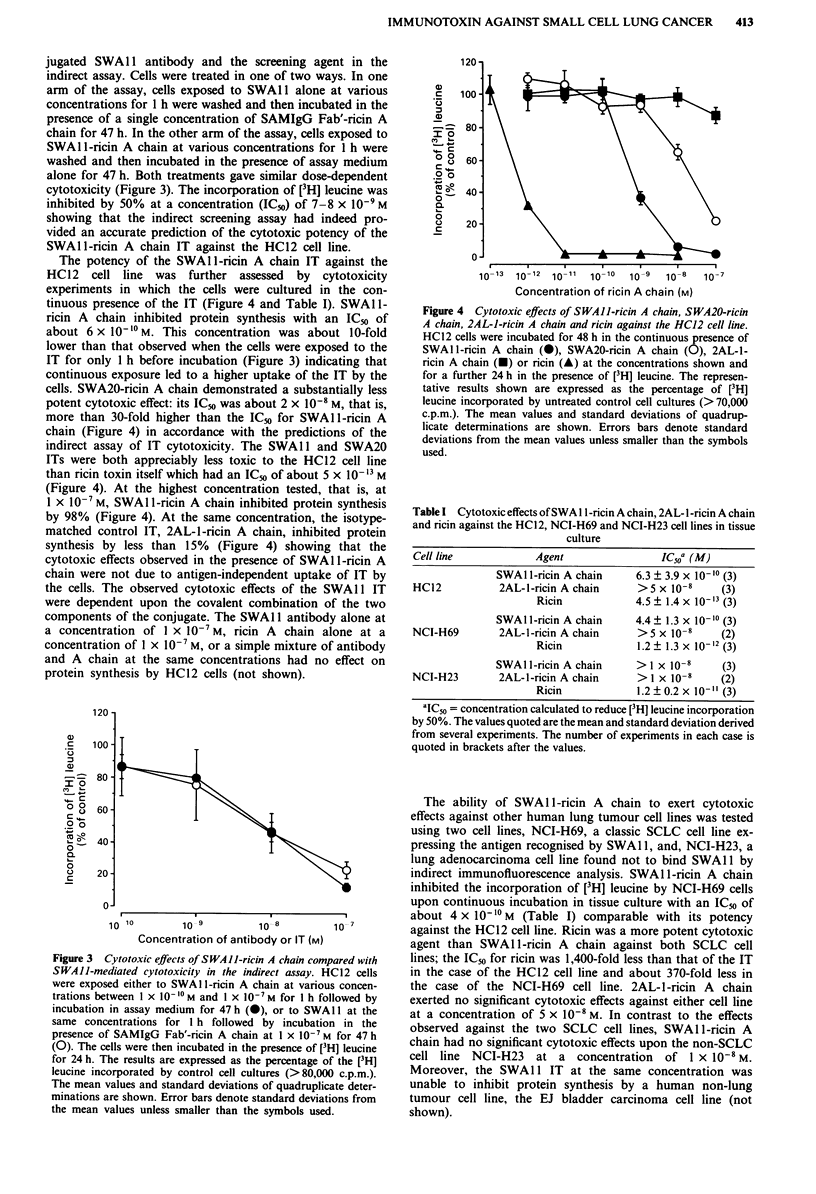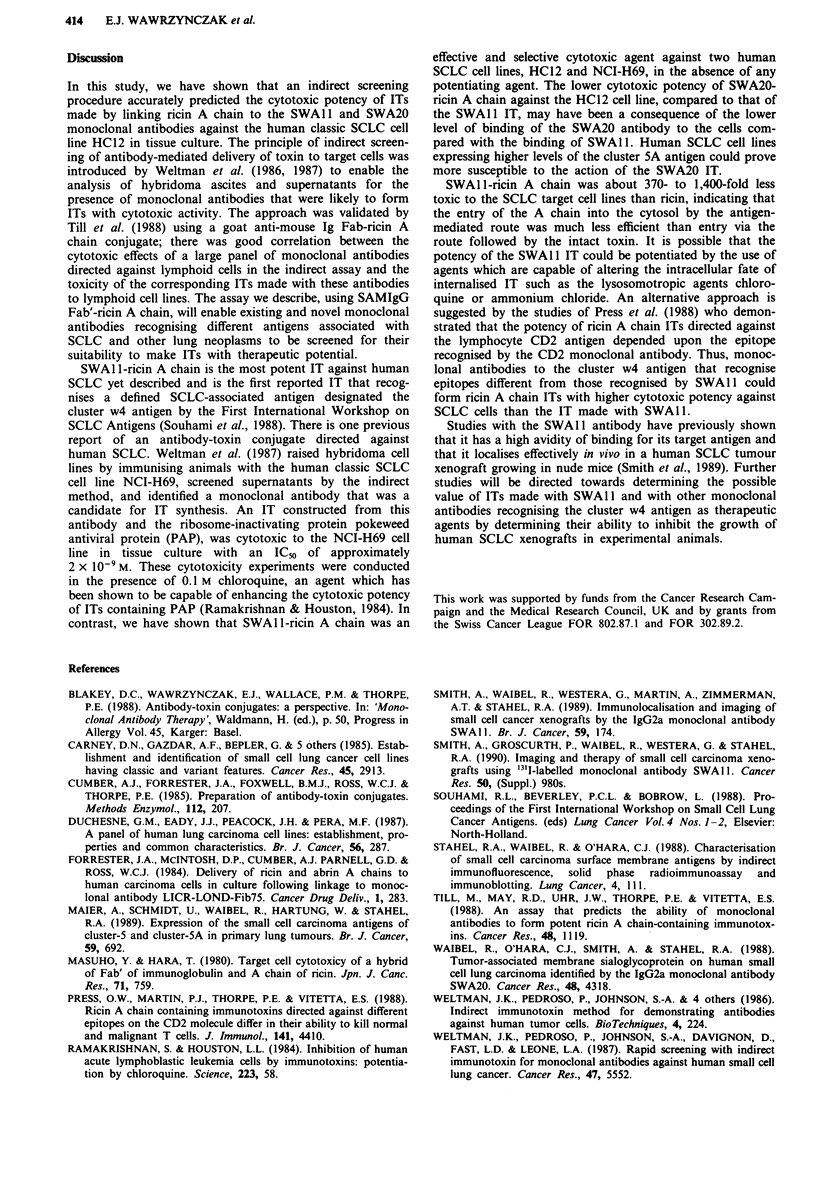# Selective cytotoxic effects of a ricin A chain immunotoxin made with the monoclonal antibody SWA11 recognising a human small cell lung cancer antigen.

**DOI:** 10.1038/bjc.1990.308

**Published:** 1990-09

**Authors:** E. J. Wawrzynczak, E. J. Derbyshire, R. V. Henry, G. D. Parnell, A. Smith, R. Waibel, R. A. Stahel

**Affiliations:** Drug Targeting Laboratory, Institute of Cancer Research, Sutton, Surrey, UK.

## Abstract

The potential of mouse monoclonal antibodies for recognising different antigens associated with human small cell lung cancer (SCLC) to form active immunotoxins was assessed by an indirect in vitro screening assay. The screening agent used was a conjugate made by linking ricin A chain to a sheep anti-mouse IgG Fab' fragment via a disulphide bond. The monoclonal antibodies SWA11 and SWA20 both mediated the toxic effects of ricin A chain against the HC12 classic SCLC cell line in dose-dependent fashion. The SWA11 antibody was the more effective; in combination with the screening agent at a concentration of 1 x 10(-7) M, it inhibited the incorporation of [3H] leucine into HC12 cells by 94% compared with only 44% inhibition in the case of SWA20. An immunotoxin made by the direct chemical conjugation of ricin A chain to SWA11 exhibited selective toxic effects upon HC12 cells in tissue culture inhibiting the incorporation of [3H] leucine by 50% at a concentration (IC50) of 6.2 x 10(-10) M and by 98% at 1 x 10(-7) M. SWA11-ricin A chain had an IC50 of 4.4 x 10(-10) M against the NCI-H69 classic SCLC cell line but showed no cytotoxic activity against the human lung adenocarcinoma cell line NCI-H23 at a concentration of 1 x 10(-8) M.


Br. J. Cancer (1990), 62, 410-414 © Macmillan Press Ltd., 1990

### Selective cytotoxic effects of a ricin A chain immunotoxin made with the monoclonal antibody SWA11 recognising a human small cell lung cancer antigen

E.J. Wawrzynczak[1], E.J. Derbyshire[1], R.V. Henry[1], G.D. Parnell[1], A. Smith[2], R. Waibel[2] & R.A. Stahel[2]

[1]*Drug Targeting Laboratory, Section of Medicine, Institute of Cancer Research, Sutton, Surrey SM2 5NG, UK; and* [2]*Division of Oncology, Department of Medicine, University Hospital, CH-8091 Zurich, Switzerland.*

**Summary** The potential of mouse monoclonal antibodies for recognising different antigens associated with human small cell lung cancer (SCLC) to form active immunotoxins was assessed by an indirect *in vitro* screening assay. The screening agent used was a conjugate made by linking ricin A chain to a sheep anti-mouse IgG Fab' fragment via a disulphide bond. The monoclonal antibodies SWA11 and SWA20 both mediated the toxic effects of ricin A chain against the HC12 classic SCLC cell line in dose-dependent fashion. The SWA11 antibody was the more effective; in combination with the screening agent at a concentration of $1 \times 10^{-7}$ M, it inhibited the incorporation of [$^3$H] leucine into HC12 cells by 94% compared with only 44% inhibition in the case of SWA20. An immunotoxin made by the direct chemical conjugation of ricin A chain to SWA11 exhibited selective toxic effects upon HC12 cells in tissue culture inhibiting the incorporation of [$^3$H] leucine by 50% at a concentration ($IC_{50}$) of $6.2 \times 10^{-10}$M and by 98% at $1 \times 10^{-7}$M. SWA11-ricin A chain had an $IC_{50}$ of $4.4 \times 10^{-10}$M against the NCI-H69 classic SCLC cell line but showed no cytotoxic activity against the human lung adenocarcinoma cell line NCI-H23 at a concentration of $1 \times 10^{-8}$M.

Lung cancer is now the most common malignancy in men world-wide and is expected to overtake breast cancer as the leading cause of mortality in women. Small cell lung cancer (SCLC), which accounts for about 25% of new cases, differs from other lung neoplasms in two important respects. Firstly, SCLC is highly metastatic. Most patients present with mediastinal metastases, extrathoracic metastases, or both. Secondly, SCLC is highly chemoresponsive. Combination chemotherapy results in high remission rates but most patients die of their disease within two years following the development of drug resistance and few patients achieve long-term survival. There is a clear need, therefore, to investigate cytotoxic agents which act by a different mechanism from the agents in current use and which can be administered systemically. A novel approach is the use of monoclonal antibodies to deliver toxic proteins, such as the ribosome-inactivating protein ricin A chain, to sites of tumour. Antibody-toxin conjugates, or immunotoxins (ITs), made with monoclonal antibodies recognising tumour-associated antigens have demonstrated selective toxic effects against human tumour cells in tissue culture and in animal models of cancer, and are currently undergoing clinical trials in patients (Blakey *et al.*, 1988).

The First International Workshop on SCLC Antigens identified several different groups, or clusters, of monoclonal antibodies that recognise discrete cell-surface antigens associated with SCLC. Each cluster of antibodies displayed a characteristic spectrum of binding to normal and malignant tissues and cell lines of pulmonary, neuroendocrine and epithelial origin (Souhami *et al.*, 1988). A number of monoclonal antibodies making up clusters designated w4 and 5A have been described previously (Stahel *et al.*, 1988). SWA11, which is associated with cluster w4, shows strong reactivity with both classic and variant SCLC cell lines *in vitro*. This antibody localises efficiently in human SCLC tumour xenografts *in vivo* (Smith *et al.*, 1989) and inhibits tumour growth when conjugated to $^{131}$I (Smith *et al.*, 1990). SWA20, belonging to cluster 5A, recognises a sialoglycoprotein antigen expressed selectively by a proportion of SCLC cell lines and primary SCLC tumours (Waibel *et al.*, 1988; Maier *et al.*, 1989).

Correspondence: E.J. Wawrzynczak.
Received 24 January 1990; and in revised form 20 April 1990.

In this study, we have assessed the potential of the anti-SCLC antibodies to form cytotoxic agents with ricin A chain by using a modified version of the indirect *in vitro* screening procedure developed by Weltman *et al.* (1987) and Till *et al.* (1988). When the human classic SCLC cell line HC12 was exposed to the SWA11 antibody and then incubated with the screening agent, sheep anti-mouse IgG Fab' fragment linked to ricin A chain (SAMIgG Fab'-ricin A chain), cellular protein synthesis was inhibited in dose-dependent fashion. An IT made by the direct chemical conjugation of ricin A chain to the SWA11 antibody displayed selective toxic effects upon the HC12 cell line, confirming the prediction of the indirect assay.

### Materials and methods

### *Materials*

Castor bean cake derived from the seeds of *Ricinus communis* of Sri Lankan origin was a gift of Croda Premier Oils, Hull, Humberside, England. The anti-SCLC mouse monoclonal antibodies, SWA11 and SWA20, were purified from hybridoma supernatants as described by Smith *et al.* (1989). The control mouse monoclonal antibody raised against vesicular stomatitis virus, 2AL-1, was purified from ascitic fluid (Forrester *et al.*, 1984). All three antibodies are of the IgG2a subclass. The human classic SCLC cell line HC12 (Duchesne *et al.*, 1987) was the gift of Dr. G. Duchesne, Institute of Cancer Research, Sutton. The human classic SCLC cell line NCI-H69 and the human lung adenocarcinoma cell line NCI-H23 (Carney *et al.*, 1985) were kindly provided by Dr. V. Macaulay, Institute of Cancer Research, Sutton.

Tissue culture medium RPMI-1640 and fetal calf serum (FCS) were purchased from Gibco Ltd., Paisley, Scotland. The 96-well and 24-well sterile tissue culture plates (Nunclon) and flat-bottomed 96-well mircoelisa plates (Immulon 2) were obtained from Dynatech Laboratories Ltd., Billingshurst, Sussex, England.

Sephadex G25(SF), Sephacryl S200(HR), Blue Sepharose CL-6B and N-succinimidyl 3-(2-pyridyldithio) propionate (SPDP) were purchased from Pharmacia Ltd., Milton Keynes, Bucks, England. Streptavidin-biotinylated horseradish peroxidase (HRP) complex (RPN. 1051) and L-[4, 5-$^3$H] leucine (TRK. 170) were from Amersham International plc, Amersham, Bucks, England. (Sheep) Anti-mouse

IgG F(ab')$_2$ fragment (M-1522), dithiothreitol (DTT), o-phenylenediamine and thimerosal were from Sigma Chemical Co. Ltd., Poole, Dorset, England. N-iodoacetyl-N-biotinylhexylenediamine (iodoacetyl-LC-biotin) was from Pierce & Warriner (UK) Ltd., Chester, Cheshire, England. 5,5'-dithiobis (2-nitrobenzoic acid) (Ellman's reagent) was purchased from Aldrich Chemical Co. Ltd., Gillingham, Dorset, England. Casein (hammersten grade) was obtained from BDH Ltd., Poole, Dorset, England. All other reagents were of the highest available purity.

*Preparation of immunotoxins:*

*Purification of ricin A chain* Ricin was isolated from an aqueous extract of defatted castor bean cake using the methods described by Cumber *et al.* (1985). Ricin A chain was isolated from the toxin by reductive cleavage and further purified using immobilised asialofetuin as described by Forrester *et al.* (1984).

*Preparation of immunotoxins with mouse monoclonal antibodies* Ricin A chain was attached to the SWA11 and SWA20 monoclonal antibodies via a disulphide bond using procedures described in detail by Cumber *et al.* (1985). Briefly, 2-pyridyl disulphide groups were introduced into SWA11 and SWA20 at an average modification level of about 1.5 groups per antibody by reaction with the SPDP reagent. The antibodies so derived were reacted overnight with an excess of freshly reduced ricin A chain. In each case, the reaction mixture was then applied to a column of Sephacryl S200 (HR) and the material which eluted at a position corresponding to a relative molecular mass ($M_r$) of approximately 180,000–210,000 was pooled. Analysis by sodium dodecyl sulphate-polyacrylamide gel electrophoresis (SDS-PAGE) indicated that the predominant IT species in these preparations contained one molecule of ricin A chain linked to one molecule of antibody. The preparations also contained smaller amounts of more highly substituted conjugate molecules and uncoupled antibody. The preparation of 2AL-1-ricin A chain by a similar procedure has been described previously (Forrester *et al.*, 1984).

*Preparation of SAMIgG Fab'-ricin A chain* SAMIgG Fab'-ricin A chain was synthesised by a method based on the work of Masuho & Hara (1980) and Till *et al.* (1988). The F (ab')$_2$ fragment of SAMIgG was dissolved to give a final concentration of about 1 mg ml$^{-1}$ in 20 mM Tris-HCl buffer containing 0.14 M NaCl, 2 mM EDTA, pH 8.2 (TE buffer). 2-Mercaptoethanol (as a 20 mM solution in TE buffer) was added to a final concentration of 2 mM and the mixture was incubated for 1 h at 37°C. Ellman's reagent (as a 50 mM solution in TE buffer) was then added to a final concentration of 5 mM and the mixture incubated for 1 h at room temperature. The mixture was then dialysed three times against 4 l of 20 mM sodium phosphate buffer containing 0.14 M NaCl, 1 mM EDTA, pH 6.5 at 4°C . To 7 ml of the solution containing about 5 mg of Fab' substituted with Ellman's reagent was added 7 ml of a solution containing 7.5 mg ricin A chain in 20 mM sodium phosphate buffer containing 0.14 M NaCl, 1 mM EDTA, pH 7.5 (PE buffer). The reaction mixture was left overnight at room temperature, then concentrated to about 8 ml by ultrafiltration using an Amicon PM10 membrane and subjected to gel permeation chromatography on a column (90 cm × 1.6 cm diameter) of Sephacryl S200 (HR) equilibrated with PE buffer. The pooled fraction corresponding to Fab'-ricin A chain ($M_r$, approximately 80,000) was concentrated to about 4 ml by ultrafiltration, dialysed into 50 mM sodium phosphate buffer, pH 7.5, and applied to a column (17 cm × 1.2 cm diameter) of Blue Sepharose CL-6B equilibrated with the same buffer. The Fab'-ricin A chain eluted from the column with 50 mM sodium phosphate buffer containing 0.5 M NaCl, pH 7.5, and was judged by SDS-PAGE to be essentially free from contamination by free ricin A chain and unconjugated antibody fragments. The product was sterilised by filtration and stored at 4°C.

*Measurement of SAMIgG Fab' binding to mouse monoclonal antibodies*

*Synthesis of SAMIgG Fab'-LC-biotin* SAMIgG F (ab')$_2$ at about 1 mg ml$^{-1}$ in TE buffer was treated with DTT at a final concentration of 10 mM for 2 h at room temperature. The reduced Fab' fragment was purified free from excess reducing agent by gel filtration on a column of Sephadex G25 (SF) equilibrated with $N_2$-flushed TE buffer. A 20-fold molar excess of N-iodoacetyl-LC-biotin, prepared as a solution in dimethylformamide at 7.6 mg ml$^{-1}$, was added and the reaction allowed to proceed for 1 h at room temperature. SAMIgG Fab'-LC-biotin was then isolated by gel filtration on a column of Sephacryl S200 (HR) equilibrated with PE buffer.

*Enzyme-linked immunosorbent assay* Mouse monoclonal antibody was adsorbed to microelisa plates by incubating each well with 100 µl of antibody solution, prepared at different concentrations in 15 mM sodium carbonate/35 mM sodium bicarbonate buffer, pH 9.6, overnight at 4°C in a humidified atmosphere. The subsequent steps were carried out at room temperature. The plates were washed four times with casein buffer (10 mM Tris-HCl, pH 7.6 containing 0.15 M NaCl, 0.5% (w/v) casein and 0.02% (w/v) thimerosal) using a Dynatech 'Miniwash' plate washer. Wells were incubated in casein buffer for 30 min and the buffer was then removed. SAMIgG Fab'-LC-biotin in casein buffer at a concentration of 1 µg ml$^{-1}$ was then added to the wells of the plate in 100 µl volumes. The plates were incubated for 2 h. After washing four times with casein buffer as above, 100 µl of streptavidin-biotinylated HRP complex diluted 1:1000 in casein buffer was added to each well for 1 h. The plates were washed three times with casein buffer and once with phosphate-buffered saline (PBS). The wells were then incubated with 100 µl of o-phenylenediamine solution (40 mg in 100 ml 52 mM $Na_2HPO_4$/24 mM citric acid containing 20 µl 100 vols. $H_2O_2$) for 10 min. The development of colour was stopped by the addition of 50 µl 12.5% (v/v) $H_2SO_4$. The plates were read at 492 nm on an automated plate reader.

*Cytotoxicity experiments in tissue culture*

The human SCLC cell lines HC12 and NCI-H69, growing in the form of multicellular spheroids in suspension, were routinely maintained in RPMI-1640 supplemented with 1% (v/v) 0.24 M glutamine solution and 10% (v/v) FCS (heat-inactivated) in a humidified atmosphere of 5% (v/v) $CO_2$ in air at 37°C. For cytotoxicity assays, the spheroids were first suspended in PBS and then disaggregated mechanically by repeated passage through a 0.6 mm gauge syringe needle to produce a suspension consisting predominantly of single cells.

In indirect assays of IT cytotoxicity, samples of mouse monoclonal antibody in PBS were distributed in 0.1 ml volumes into the wells of a 96-well tissue culture plate. The HC12 singled cell suspension, 0.1 ml containing 1 × 10$^5$ cells, was added to each well and incubated for 1 h at 4°C. The cells were then washed once with cold PBS and resuspended in 0.2 ml of leucine-free RPMI-1640/10% FCS/2.4 mM glutamine (assay medium) alone, or in assay medium containing SAMIgG FAb'-ricin A chain. Following incubation for 47 h at 37°C, 1 µCi of [$^3$H] leucine was added to each well and the cultures were incubated for a further 24 h at 37°C before the cells were harvested on filters using a Titertek automated cell harvester. The incorporation of [$^3$H] leucine was determined by liquid scintillation counting of the filters in an LKB Rackbeta Liquid Scintillation Counter. In continuous assays of cytotoxic activity using the HC12 and NCI-H69 cell lines, 0.1 ml samples of antibody, IT or ricin prepared at different concentrations in assay medium, were mixed with 0.1 ml of a singled cell suspension and the cells were incubated without washing for 48 h at 37°C before pulsing with [$^3$H] leucine for 24 h, harvesting and counting of radioactivity as described above. All assays were performed in quadruplicate.

The human lung adenocarcinoma cell line NCI-H23 was routinely maintained in RPMI-1640/10% FCS/2.4 mM glutamine in a humidified atmosphere of 5% (v/v) $CO_2$ in air at 37°C. Cells were harvested with trypsin/versene, washed with fresh medium, and distributed in 1 ml volumes into the wells of a 24-well sterile tissue culture plate to give a total of $1 \times 10^5$ cells per well. The plates were incubated for 48 h at 37°C to allow the cells to adhere to the plate. The medium was removed by aspiration and replaced with 0.9 ml assay medium. Samples (0.1 ml) of IT or ricin at different concentrations in assay medium were then added to cell cultures in triplicate and the plates incubated for 24 h. [$^3$H] Leucine at 1 μCi per well was added and the incubation continued for a further 24 h. The cells were washed three times with PBS, fixed once with 5% (w/v) trichloroacetic acid, washed with methanol and dried. The content of each well was solubilised by incubation with 0.2 ml of 1 M NaOH for 1 h at 37°C. The incorporation of [$^3$H] leucine was determined by liquid scintillation counting of 0.15 ml samples of the solubilised cellular contents.

### Results

### *Indirect assay of immunotoxin cytotoxicity*

The ability of the two anti-SCLC monoclonal antibodies, SWA11 and SWA20, to mediate ricin A chain toxicity against the human classic SCLC cell line HC12 was tested by an indirect screening assay *in vitro*. Both monoclonal antibodies bound to a high proportion of HC12 cells by indirect immunofluorescence analysis, SWA11 giving the greater intensity of fluorescence staining on cells treated with the antibodies at identical concentration. A third monoclonal antibody, 2AL-1, found not to bind to HC12 cells by indirect immunofluorescence analysis, was included as a negative control. The binding of the SAMIgG Fab' fragment to the three mouse monoclonal antibodies was measured by an enzyme-linked immunosorbent assay using the biotinylated Fab' fragment. SAMIgG Fab'-LC-biotin bound to immobilised mouse antibody was detected using a streptavidin-biotinylated HRP complex. As shown in Figure 1, there was no significant difference between the binding curves for the three monoclonal antibodies, indicating that the Fab' fragment bound similarly to each antibody.

In the indirect assay of IT cytotoxicity, HC12 cells were first treated with a single concentration of each of the three monoclonal antibodies. The cells were then washed and incubated with the screening agent, SAMIgG Fab'-ricin A chain, at various concentrations. Figure 2 shows the effects of these treatments on cellular protein synthesis. The incorporation of [$^3$H] leucine was greatly reduced in cells treated with SWA11 followed by the screening agent, compared with cells in control cultures incubated with the screening agent alone. This cytotoxic effect was dose-dependent; protein synthesis was inhibited by 75% at a SAMIgG Fab'-ricin A chain concentration of $1 \times 10^{-8}$ M and by 94% at the 10-fold higher concentration of the screening agent. The inhibition of protein synthesis was dependent upon the presence of the screening agent because cells exposed to the SWA11 antibody alone were not affected. Protein synthesis in HC12 cells treated with SWA20 in combination with the screening agent was also inhibited in a dose-dependent manner. However, the effect was much weaker than that observed with SWA11; there was no significant inhibition of [$^3$H] leucine incorporation at a SAMIgG Fab'-ricin A chain concentration of $1 \times 10^{-8}$ M and protein synthesis was inhibited by only 44% with the screening agent at a concentration of $1 \times 10^{-7}$ M. No significant effects were observed when the non-binding antibody, 2AL-1, was tested in the assay in combination with the screening agent. The results of the indirect assay indicated that, although the screening agent bound equally well to all three monoclonal antibodies, only the antibodies able to bind to the HC12 cell line demonstrated cytotoxic effects. The SWA11 antibody was the most effective at mediating the entry into the cells of ricin A chain delivered by the SAMIgG Fab' fragment. This finding strongly suggested that an IT made by linking ricin A chain directly to the SWA11 antibody would exert comparable cytotoxic effects upon HC12 cells and other cells expressing the antigen recognised by SWA11.

**Figure 1** *Binding of SAMIgG Fab' fragment to mouse monoclonal antibodies.* Microelisa plates treated with SWA11 (●), SWA20 (○), or 2AL-1 (■) at various concentrations between 0.01 and 0.1 μg ml$^{-1}$, were exposed to a single concentration (1 μg ml$^{-1}$) of SAMIgG Fab'-LC-biotin and then to streptavidin-biotinylated HRP complex. The absorbance at 492 nm developed by the substrate solution was taken as a measure of the amount of the Fab' fragment bound. Each point shown represents the arithmetic mean value of sextuplicate determinations. The error bars denote the standard deviations from the mean values unless smaller than the symbols used.

**Figure 2** *Indirect assay of immunotoxin cytotoxicity.* HC12 cells exposed to SWA11 (●), SWA20 (○), or 2AL-1 (■) at a final concentration of 25 μg ml$^{-1}$ for 1 h, were washed, incubated in the presence of SAMIgG Fab'-ricin A chain at $1 \times 10^{-8}$ M, $2.5 \times 10^{-8}$ M and $1 \times 10^{-7}$ M for 48 h and then incubated in the presence of [$^3$H] leucine for a further 24 h. The effects of these treatments are expressed as the percentage of the [$^3$H] leucine incorporated by untreated control cell cultures (> 100,000 c.p.m.). Each point shown represents the arithmetic mean value of quadruplicate determinations of [$^3$H] leucine incorporation. The error bars denote the standard deviations from the mean values unless smaller than the symbols used. The [$^3$H] leucine incorporation of control cultures incubated with SAMIgG Fab'-ricin A chain alone at $1 \times 10^{-7}$ M was inhibited by less than 20%.

### *Cytotoxic activity of ricin A chain immunotoxins made with SWA11, SWA20 and 2AL-1*

The toxic effect of treating HC12 cells with an IT made by linking ricin A chain directly to the SWA11 antibody was compared with the effect given by combination of the unconjugated SWA11 antibody and the screening agent in the indirect assay. Cells were treated in one of two ways. In one arm of the assay, cells exposed to SWA11 alone at various concentrations for 1 h were washed and then incubated in the presence of a single concentration of SAMIgG Fab'-ricin A chain for 47 h. In the other arm of the assay, cells exposed to SWA11-ricin A chain at various concentrations for 1 h were washed and then incubated in the presence of assay medium alone for 47 h. Both treatments gave similar dose-dependent cytotoxicity (Figure 3). The incorporation of [$^3$H] leucine was inhibited by 50% at a concentration ($IC_{50}$) of 7–8 × $10^{-9}$ M showing that the indirect screening assay had indeed provided an accurate prediction of the cytotoxic potency of the SWA11-ricin A chain IT against the HC12 cell line.

The potency of the SWA11-ricin A chain IT against the HC12 cell line was further assessed by cytotoxicity experiments in which the cells were cultured in the continuous presence of the IT (Figure 4 and Table I). SWA11-ricin A chain inhibited protein synthesis with an $IC_{50}$ of about 6 × $10^{-10}$ M. This concentration was about 10-fold lower than that observed when the cells were exposed to the IT for only 1 h before incubation (Figure 3) indicating that continuous exposure led to a higher uptake of the IT by the cells. SWA20-ricin A chain demonstrated a substantially less potent cytotoxic effect: its $IC_{50}$ was about 2 × $10^{-8}$ M, that is, more than 30-fold higher than the $IC_{50}$ for SWA11-ricin A chain (Figure 4) in accordance with the predictions of the indirect assay of IT cytotoxicity. The SWA11 and SWA20 ITs were both appreciably less toxic to the HC12 cell line than ricin toxin itself which had an $IC_{50}$ of about 5 × $10^{-13}$ M (Figure 4). At the highest concentration tested, that is, at 1 × $10^{-7}$ M, SWA11-ricin A chain inhibited protein synthesis by 98% (Figure 4). At the same concentration, the isotype-matched control IT, 2AL-1-ricin A chain, inhibited protein synthesis by less than 15% (Figure 4) showing that the cytotoxic effects observed in the presence of SWA11-ricin A chain were not due to antigen-independent uptake of IT by the cells. The observed cytotoxic effects of the SWA11 IT were dependent upon the covalent combination of the two components of the conjugate. The SWA11 antibody alone at a concentration of 1 × $10^{-7}$ M, ricin A chain alone at a concentration of 1 × $10^{-7}$ M, or a simple mixture of antibody and A chain at the same concentrations had no effect on protein synthesis by HC12 cells (not shown).

**Figure 3** *Cytotoxic effects of SWA11-ricin A chain compared with SWA11-mediated cytotoxicity in the indirect assay.* HC12 cells were exposed either to SWA11-ricin A chain at various concentrations between 1 × $10^{-10}$ M and 1 × $10^{-7}$ M for 1 h followed by incubation in assay medium for 47 h (●), or to SWA11 at the same concentrations for 1 h followed by incubation in the presence of SAMIgG Fab'-ricin A chain at 1 × $10^{-7}$ M for 47 h (○). The cells were then incubated in the presence of [$^3$H] leucine for 24 h. The results are expressed as the percentage of the [$^3$H] leucine incorporated by control cell cultures (> 80,000 c.p.m.). The mean values and standard deviations of quadruplicate determinations are shown. Error bars denote standard deviations from the mean values unless smaller than the symbols used.

**Figure 4** *Cytotoxic effects of SWA11-ricin A chain, SWA20-ricin A chain, 2AL-1-ricin A chain and ricin against the HC12 cell line.* HC12 cells were incubated for 48 h in the continuous presence of SWA11-ricin A chain (●), SWA20-ricin A chain (○), 2AL-1-ricin A chain (■) or ricin (▲) at the concentrations shown and for a further 24 h in the presence of [$^3$H] leucine. The representative results shown are expressed as the percentage of [$^3$H] leucine incorporated by untreated control cell cultures (> 70,000 c.p.m.). The mean values and standard deviations of quadruplicate determinations are shown. Errors bars denote standard deviations from the mean values unless smaller than the symbols used.

**Table I** Cytotoxic effects of SWA11-ricin A chain, 2AL-1-ricin A chain and ricin against the HC12, NCI-H69 and NCI-H23 cell lines in tissue culture

| *Cell line* | *Agent* | *$IC_{50}$[a] (M)* |
|---|---|---|
| | SWA11-ricin A chain | 6.3 ± 3.9 × $10^{-10}$ (3) |
| HC12 | 2AL-1-ricin A chain | > 5 × $10^{-8}$ (3) |
| | Ricin | 4.5 ± 1.4 × $10^{-13}$ (3) |
| | SWA11-ricin A chain | 4.4 ± 1.3 × $10^{-10}$ (3) |
| NCI-H69 | 2AL-1-ricin A chain | > 5 × $10^{-8}$ (2) |
| | Ricin | 1.2 ± 1.3 × $10^{-12}$ (3) |
| | SWA11-ricin A chain | > 1 × $10^{-8}$ (3) |
| NCI-H23 | 2AL-1-ricin A chain | > 1 × $10^{-8}$ (2) |
| | Ricin | 1.2 ± 0.2 × $10^{-11}$ (3) |

[a]$IC_{50}$ = concentration calculated to reduce [$^3$H] leucine incorporation by 50%. The values quoted are the mean and standard deviation derived from several experiments. The number of experiments in each case is quoted in brackets after the values.

The ability of SWA11-ricin A chain to exert cytotoxic effects against other human lung tumour cell lines was tested using two cell lines, NCI-H69, a classic SCLC cell line expressing the antigen recognised by SWA11, and, NCI-H23, a lung adenocarcinoma cell line found not to bind SWA11 by indirect immunofluorescence analysis. SWA11-ricin A chain inhibited the incorporation of [$^3$H] leucine by NCI-H69 cells upon continuous incubation in tissue culture with an $IC_{50}$ of about 4 × $10^{-10}$ M (Table I) comparable with its potency against the HC12 cell line. Ricin was a more potent cytotoxic agent than SWA11-ricin A chain against both SCLC cell lines; the $IC_{50}$ for ricin was 1,400-fold less than that of the IT in the case of the HC12 cell line and about 370-fold less in the case of the NCI-H69 cell line. 2AL-1-ricin A chain exerted no significant cytotoxic effects against either cell line at a concentration of 5 × $10^{-8}$ M. In contrast to the effects observed against the two SCLC cell lines, SWA11-ricin A chain had no significant cytotoxic effects upon the non-SCLC cell line NCI-H23 at a concentration of 1 × $10^{-8}$ M. Moreover, the SWA11 IT at the same concentration was unable to inhibit protein synthesis by a human non-lung tumour cell line, the EJ bladder carcinoma cell line (not shown).

### Discussion

In this study, we have shown that an indirect screening procedure accurately predicted the cytotoxic potency of ITs made by linking ricin A chain to the SWA11 and SWA20 monoclonal antibodies against the human classic SCLC cell line HC12 in tissue culture. The principle of indirect screening of antibody-mediated delivery of toxin to target cells was introduced by Weltman *et al.* (1986, 1987) to enable the analysis of hybridoma ascites and supernatants for the presence of monoclonal antibodies that were likely to form ITs with cytotoxic activity. The approach was validated by Till *et al.* (1988) using a goat anti-mouse Ig Fab-ricin A chain conjugate; there was good correlation between the cytotoxic effects of a large panel of monoclonal antibodies directed against lymphoid cells in the indirect assay and the toxicity of the corresponding ITs made with these antibodies to lymphoid cell lines. The assay we describe, using SAMIgG Fab'-ricin A chain, will enable existing and novel monoclonal antibodies recognising different antigens associated with SCLC and other lung neoplasms to be screened for their suitability to make ITs with therapeutic potential.

SWA11-ricin A chain is the most potent IT against human SCLC yet described and is the first reported IT that recognises a defined SCLC-associated antigen designated the cluster w4 antigen by the First International Workshop on SCLC Antigens (Souhami *et al.*, 1988). There is one previous report of an antibody-toxin conjugate directed against human SCLC. Weltman *et al.* (1987) raised hybridoma cell lines by immunising animals with the human classic SCLC cell line NCI-H69, screened supernatants by the indirect method, and identified a monoclonal antibody that was a candidate for IT synthesis. An IT constructed from this antibody and the ribosome-inactivating protein pokeweed antiviral protein (PAP), was cytotoxic to the NCI-H69 cell line in tissue culture with an $IC_{50}$ of approximately $2 \times 10^{-9}$ M. These cytotoxicity experiments were conducted in the presence of 0.1 M chloroquine, an agent which has been shown to be capable of enhancing the cytotoxic potency of ITs containing PAP (Ramakrishnan & Houston, 1984). In contrast, we have shown that SWA11-ricin A chain was an effective and selective cytotoxic agent against two human SCLC cell lines, HC12 and NCI-H69, in the absence of any potentiating agent. The lower cytotoxic potency of SWA20-ricin A chain against the HC12 cell line, compared to that of the SWA11 IT, may have been a consequence of the lower level of binding of the SWA20 antibody to the cells compared with the binding of SWA11. Human SCLC cell lines expressing higher levels of the cluster 5A antigen could prove more susceptible to the action of the SWA20 IT.

SWA11-ricin A chain was about 370- to 1,400-fold less toxic to the SCLC target cell lines than ricin, indicating that the entry of the A chain into the cytosol by the antigen-mediated route was much less efficient than entry via the route followed by the intact toxin. It is possible that the potency of the SWA11 IT could be potentiated by the use of agents which are capable of altering the intracellular fate of internalised IT such as the lysosomotropic agents chloroquine or ammonium chloride. An alternative approach is suggested by the studies of Press *et al.* (1988) who demonstrated that the potency of ricin A chain ITs directed against the lymphocyte CD2 antigen depended upon the epitope recognised by the CD2 monoclonal antibody. Thus, monoclonal antibodies to the cluster w4 antigen that recognise epitopes different from those recognised by SWA11 could form ricin A chain ITs with higher cytotoxic potency against SCLC cells than the IT made with SWA11.

Studies with the SWA11 antibody have previously shown that it has a high avidity of binding for its target antigen and that it localises effectively *in vivo* in a human SCLC tumour xenograft growing in nude mice (Smith *et al.*, 1989). Further studies will be directed towards determining the possible value of ITs made with SWA11 and with other monoclonal antibodies recognising the cluster w4 antigen as therapeutic agents by determining their ability to inhibit the growth of human SCLC xenografts in experimental animals.

This work was supported by funds from the Cancer Research Campaign and the Medical Research Council, UK and by grants from the Swiss Cancer League FOR 802.87.1 and FOR 302.89.2.

### References

BLAKEY, D.C., WAWRZYNCZAK, E.J., WALLACE, P.M. & THORPE, P.E. (1988). Antibody-toxin conjugates: a perspective. In: *'Monoclonal Antibody Therapy'*, Waldmann, H. (ed.), p. 50, Progress in Allergy Vol. 45, Karger: Basel.

CARNEY, D.N., GAZDAR, A.F., BEPLER, G. & 5 others (1985). Establishment and identification of small cell lung cancer cell lines having classic and variant features. *Cancer Res.*, **45**, 2913.

CUMBER, A.J., FORRESTER, J.A., FOXWELL, B.M.J., ROSS, W.C.J. & THORPE, P.E. (1985). Preparation of antibody-toxin conjugates. *Methods Enzymol.*, **112**, 207.

DUCHESNE, G.M., EADY, J.J., PEACOCK, J.H. & PERA, M.F. (1987). A panel of human lung carcinoma cell lines: establishment, properties and common characteristics. *Br. J. Cancer*, **56**, 287.

FORRESTER, J.A., MCINTOSH, D.P., CUMBER, A.J. PARNELL, G.D. & ROSS, W.C.J. (1984). Delivery of ricin and abrin A chains to human carcinoma cells in culture following linkage to monoclonal antibody LICR-LOND-Fib75. *Cancer Drug Deliv.*, **1**, 283.

MAIER, A., SCHMIDT, U., WAIBEL, R., HARTUNG, W. & STAHEL, R.A. (1989). Expression of the small cell carcinoma antigens of cluster-5 and cluster-5A in primary lung tumours. *Br. J. Cancer*, **59**, 692.

MASUHO, Y. & HARA, T. (1980). Target cell cytotoxicy of a hybrid of Fab' of immunoglobulin and A chain of ricin. *Jpn. J. Canc. Res.*, **71**, 759.

PRESS, O.W., MARTIN, P.J., THORPE, P.E. & VITETTA, E.S. (1988). Ricin A chain containing immunotoxins directed against different epitopes on the CD2 molecule differ in their ability to kill normal and malignant T cells. *J. Immunol.*, **141**, 4410.

RAMAKRISHNAN, S. & HOUSTON, L.L. (1984). Inhibition of human acute lymphoblastic leukemia cells by immunotoxins: potentiation by chloroquine. *Science*, **223**, 58.

SMITH, A., WAIBEL, R., WESTERA, G., MARTIN, A., ZIMMERMAN, A.T. & STAHEL, R.A. (1989). Immunolocalisation and imaging of small cell cancer xenografts by the IgG2a monoclonal antibody SWA11. *Br. J. Cancer*, **59**, 174.

SMITH, A., GROSCURTH, P., WAIBEL, R., WESTERA, G. & STAHEL, R.A. (1990). Imaging and therapy of small cell carcinoma xenografts using $^{131}$I-labelled monoclonal antibody SWA11. *Cancer Res.* **50**, (Suppl.) 980s.

SOUHAMI, R.L., BEVERLEY, P.C.L. & BOBROW, L. (1988). Proceedings of the First International Workshop on Small Cell Lung Cancer Antigens. (eds) *Lung Cancer Vol. 4 Nos. 1–2*, Elsevier: North-Holland.

STAHEL, R.A., WAIBEL, R. & O'HARA, C.J. (1988). Characterisation of small cell carcinoma surface membrane antigens by indirect immunofluorescence, solid phase radioimmunoassay and immunoblotting. *Lung Cancer*, 4, 111.

TILL, M., MAY, R.D., UHR, J.W., THORPE, P.E. & VITETTA, E.S. (1988). An assay that predicts the ability of monoclonal antibodies to form potent ricin A chain-containing immunotoxins. *Cancer Res.*, **48**, 1119.

WAIBEL, R., O'HARA, C.J., SMITH, A. & STAHEL, R.A. (1988). Tumor-associated membrane sialoglycoprotein on human small cell lung carcinoma identified by the IgG2a monoclonal antibody SWA20. *Cancer Res.*, **48**, 4318.

WELTMAN, J.K., PEDROSO, P., JOHNSON, S.-A. & 4 others (1986). Indirect immunotoxin method for demonstrating antibodies against human tumor cells. *BioTechniques*, **4**, 224.

WELTMAN, J.K., PEDROSO, P., JOHNSON, S.-A., DAVIGNON, D., FAST, L.D. & LEONE, L.A. (1987). Rapid screening with indirect immunotoxin for monoclonal antibodies against human small cell lung cancer. *Cancer Res.*, **47**, 5552.